# Reconstructing AI Ethics Principles: Rawlsian Ethics of Artificial Intelligence

**DOI:** 10.1007/s11948-024-00507-y

**Published:** 2024-10-09

**Authors:** Salla Westerstrand

**Affiliations:** Turku School of Economics, Information Systems Sciences, University of Turku, Turku, Finland

**Keywords:** Artificial intelligence, AI ethics, Rawls, Justice as fairness, Democracy

## Abstract

The popularisation of Artificial Intelligence (AI) technologies has sparked discussion about their ethical implications. This development has forced governmental organisations, NGOs, and private companies to react and draft ethics guidelines for future development of ethical AI systems. Whereas many ethics guidelines address values familiar to ethicists, they seem to lack in ethical justifications. Furthermore, most tend to neglect the impact of AI on democracy, governance, and public deliberation. Existing research suggest, however, that AI can threaten key elements of western democracies that are ethically relevant. In this paper, Rawls’s theory of justice is applied to draft a set of guidelines for organisations and policy-makers to guide AI development towards a more ethical direction. The goal is to contribute to the broadening of the discussion on AI ethics by exploring the possibility of constructing AI ethics guidelines that are philosophically justified and take a broader perspective of societal justice. The paper discusses how Rawls’s theory of justice as fairness and its key concepts relate to the ongoing developments in AI ethics and gives a proposition of how principles that offer a foundation for operationalising AI ethics in practice could look like if aligned with Rawls’s theory of justice as fairness.

## Introduction

The recent popularisation of Artificial Intelligence (AI) technologies has prompted discussion about their ethical implications. Many have speculated over the potential of AI systems to threaten societal structures and quality of life (Bostrom, 2016, 2017; Coeckelbergh, 2022a, 2024; Russell, 2019). This development has pushed organisations to steer the development and application of AI systems towards a more ethical direction through principles and guidelines (see Jobin et al., 2019; Hagendorff, 2020; Ayling & Chapman, 2022). This trend also known as principalism (Clouser & Gert, 1990) has raised questions about the applicability of these principles in practice (Hickok, 2021; Mittelstadt, 2019), which has led to an increasing volume of research about operationalisation of ethics principles (Bleher & Braun, 2023; Morley et al., 2021, 2023; Stix, 2021). Whereas research around operationalising is essential in order for the principles to effectively guide AI development and deployment, I argue that the work around actionable ethics principles needs revisiting.

Many of the principles in existing guidelines are overlapping (Ashok et al., 2022; Hagendorff, 2020; Hunkenschroer & Luetge, 2022; Jobin et al., 2019), but they also differ in ways that bring forth challenges. They seem to differ in how principles are interpreted (Jobin et al., 2019) and seem to rarely guide the reader through the reasoning leading to the principles (Franzke, 2022; Jobin et al., 2019). Whereas the reviews such as those of Jobin et al., (2019) and Hagendorff (2020) map principles and values found in the guidelines, the very use of *ethics* behind the guidelines remains obscured (Franzke, 2022). In common language, one might talk about ethical questions while referring to predefined rights and wrongs in the given normative context. As Stahl (2022) points out, such issues could be better described as *social concerns* rather than issues of ethics. An ethicist, in contrast, could ask: Why are the suggested principles the most ethical ones? If the guidelines do not offer justifications, are the guidelines truly *ethics* guidelines, or just values or opinions, or a result of a political processes? As Bleher and Braun (2023) put it, if we do not ground our principles into rigorous ethical reasoning, they and the tools for their operationalisation risk being”either inappropriate, meaningless, or merely an end in themselves” (p. 10).

Moreover, Hagendorff (2020) observed that most AI ethics guidelines he reviewed tend to neglect impacts of AI on democracy, governance, and political deliberation. Meanwhile, several studies imply that the current developments in AI might threaten democracy at large (Coeckelbergh, 2022a, 2024) and harm democratic governance by distorting political opinion formation and elections (Alnemr, 2020; Chesney & Citron, 2019; Feezell et al., 2021; König & Wenzelburger, 2020; Manheim & Kaplan, 2019; Nemitz, 2018; Paterson & Hanley, 2020), eroding trust towards democratic institutions (Chesney & Citron, 2019; Manheim & Kaplan, 2019; Paterson & Hanley, 2020), and violating fundamental democratic values, such as equality and justice (Hacker, 2018; Janssen et al., 2022; König & Wenzelburger, 2020; Tolan, 2019). As AI systems with ever broader collective impacts on societies get popularised, such as generative AI systems, a need for shift in perspective from human-in-the-loop towards society-in-the loop suggested by Rahwan (2018) gains ever more relevance. Ethics as an approach seems to have potential for increasing our understanding of the relationship between AI and democracy (Westerstrand, 2023), and Stahl also noted a connection between the ongoing AI ethics discourse with regulation, such as human rights (Stahl, 2022). This implies that to improve the state of AI systems and their alignment with collective values, it might be wise to inspect the larger ecosystem and the ethical foundations behind its guiding principles.

In this paper, I contribute to the discussion through the perspective of John Rawls’s theory of justice as fairness. Rawls intended his contractarian theory to build “the most appropriate moral basis for a democratic society” (Rawls, 1971, p. viii), which makes it a theoretically interesting foundation for discussing the ethicality of AI development that comes with an increasing collective ethical and societal implications. By taking a relatively unpopular direction and contributing to the principalist paradigm in contrast to several other applications of Rawls’s theory concentrating on algorithmic applications (e.g., Leben, 2017, 2018; Heidari et al., 2019; see also Keeling, 2018), this paper aims to strengthen the ethical rigour of the principalist discourse that keeps informing the technical development of algorithms, the spheres of policy and regulation, as well as strategic decision-making regarding which technologies we should develop and deploy in the first place, and in which contexts.

The paper begins with an overview of Rawls’s theory of justice in the context of AI, which serves as a theoretical starting point for revisiting AI ethics guidelines. Then, a suggestion is drafted for a set of ethics guidelines that is based on Rawls’s principles of justice as fairness. Finally, conclusions are drawn, the academic and practical potential of the work are discussed, and possibilities for future research and application are identified.

## AI Ethics from a Rawlsian Perspective

John Rawls established his theory of justice as fairness mostly in his books *A Theory of Justice* (first published in 1971, revised in 1999), and *Political Liberalism* (1993). He intended his contractarian theory to form the most adequate moral basis for a democratic society (Rawls, 1999, p. 6). He introduced a set of principles he argues would have been agreed upon in the fairest possible setting by neutral, mutually disinterested individuals. According to Rawls, the principles read as follows:

a) Each person has an equal right to a fully adequate scheme of equal basic liberties which is compatible with a similar scheme of liberties for all. b) Social and economic inequalities are to satisfy two conditions. First, they must be attached to offices and positions open to all under conditions of fair equality of opportunity; and second, they must be to the greatest benefit of the least advantaged members of society. (Rawls, 2005, p. 291).

These principles would form the moral basis for the institutions belonging to the basic structure of society, deciding upon basic liberties and the distribution of opportunities and inequalities. Which institutions belong to the basic structure of society is not, however, unambiguous. In academic discourse, some argue that the basic structure only includes legally coercive institutions and not private corporations (*coercive account*), whereas others suggest the defining factor to be the profoundness of the impact of an institution to people’s lives (*profound effects account*) (Berkey, 2021; see, e.g., the profound effects account by Blanc & Al-Amoudi, 2013, and the coercive account of Singer, 2015). Rawls himself only provides partially contradictory statements (e.g., Rawls, 1971, 1999, p. 6 vs. Rawls, 2005, p. 265–266), which opens the discussion for differing interpretations.

In the context of AI, the discussion is further complicated by the variety of organisations in the market, ranging from non-profits and open-source communities to large private corporations and governmental institutions, each of which come with varying roles in the AI system lifecycle (see, e.g., Barclay & Abramson, 2021). For the purposes of this paper, it suffices to establish that all organisations – whether private, public or non-profit – that provide or deploy AI systems are here considered to be subject to the principles of justice based on a) their profound impact on people’s lives, b) the way in which they limit people’s ability to act as free and equal beings, and c) their key role in securing just background conditions for people and associations to function in the society.

Characteristic of a contractarian, Rawls establishes an idea of a hypothetical *original position* to define the fairest conditions possible where the principles of justice would be agreed upon. In Rawls’s original position, the principles of justice are defined by people behind a *veil of ignorance*: they know neither their position in the society, nor their personal attributes that might affect the distribution of rights and duties (Rawls, 1999, p. 11). Even one’s conception of good is hidden, leaving the parties with knowledge only of factors that are relevant for justice (Rawls, 1999, p. 11). The parties in the original position are “rational and mutually disinterested” (Rawls, 1999, p. 12), meaning that they do not pay attention to other parties’ interests or goals, but merely “prefer more primary social goods rather than less” for themselves, thus knowing that factors such as liberties, opportunities and means to promote their aims are worth defending (Rawls, 1999, p. 123). The parties are also equal in their conception of good and capability of sense of justice (Rawls, 1999, p. 17).

Consequently, the principles of justice defined by Rawls are justified by his arguments on the fairness of the original position. In this paper, I will not start drafting the ethics guidelines by returning to the original position but start by applying the principles defined by Rawls. However, Rawls meant the original position to be such that one can always enter it again (Rawls, 1999, p. 17) and by *reflective equilibrium* adjust the contractarian situation, as well as the principles resulting from it, to eventually end up with principles that are just in a particular context (Rawls, 1999, p. 18). In the context of AI and fairness, this adjusting process has been shown to be particularly relevant (see, e.g., Franke, 2021), which also shows in the analysis below.

When Rawls first published his theory of justice, AI ethics as a field of study was in its infancy with only a couple of notable exceptions (see e.g., Wiener, 1954; Weizenbaum, 1976; see also Bynum, 2006). Hence, Rawls himself has given little indications of the applicability of his theory to the context of AI. His theory has, however, been shown to serve as a viable alternative to utilitarian methods of embedding ethics into algorithms and robotics by, e,g, Leben (2017, 2018). Taking a Rawlsian approach can also be justified by the relevance of the concept of justice in the current AI principles and guidelines (see Jobin et al., 2019). Several scholars also address justice related to biased algorithms and discrimination (Bigman et al., 2023; Hacker, 2018; Janssen et al., 2022; König & Wenzelburger, 2020; Tolan, 2019), leading to an emergence of a field called *algorithmic fairness* (Lepri et al., 2018; Mitchell et al., 2021). Therefore, it seems that justice and fairness are values that people in modern democracies strive for AI technologies being no exception—which makes Rawls’s theory of justice a theoretically interesting starting point for ethical AI principles.

Rawls’s theory has deservedly received critique. For instance, Sen discusses Rawls’s perception of justice as fairness in his *Idea of Justice* (Sen, 2010) and argues that the theory is weakly applicable to global context, where the institutions are not national or international, but supranational and global (Anderson, 2003; Harsanyi, 1975). In addition, Harsanyi (1975) has voiced critique on Rawls’s decision to use the maximin principle as a decision theory that guides the reasoning in the original position, and thus the formation of the principles of justice. Robert Nozick (2013 [1974]), on the other hand, responded to Rawls’s theory of justice with an *entitlement theory* of justice. According to Nozick, instead of aiming for an equal distribution of basic goods, everyone should get what they are entitled to, whether through justice in acquisition, transfer or rectification of justice (Nozick, 2013, p. 150–153), thus proposing a libertarian view to distributive justice. In the context of AI and algorithmic systems, embedding Rawlsian ethics into algorithms has also been challenged (e.g., critique on Leben’s work in Keeling, 2018). While several of these limitations are discussed below, a further discussion on the moral theoretical soundness of Rawls’s theory is a topic for another paper. These considerations in mind, we do not suggest this work to be the ultimate solution for fair development and deployment of AI systems but rather one contribution to the broader set of works striving towards ethical digital societies. In this discussion, Rawls still offers a useful starting point for reflecting the ethical foundations of AI in the context of democratic societies, which I demonstrate below.

## Rawlsian Ethics Guidelines for Fair AI

In what follows, I apply Rawls’s theory to form a set of ethics guidelines for fair AI. In the Rawlsian spirit, the guidelines here are destined to the basic structure of society (see Sect.  2) in which organisations develop and/or use AI systems.

### Principle 1: Basic Liberties

According to the first principle of justice, each person should have equal right to “the most extensive scheme of equal basic liberties compatible with a similar scheme of liberties for others” (Rawls, 1999, p. 53). Rawls offers a preliminary list of basic liberties in *A Theory of Justice* and further develops and justifies them in *Political Liberalism*. Accordingly, basic liberties to be equally distributed are:

- Freedom of thought and liberty of conscience
- Political liberties and freedom of association (the right to vote and to hold public office)
- Liberty and integrity of the person (including freedom from psychological oppression and physical assault and dismemberment)
- Liberties covered by the rule of law. (Rawls, 1999, p. 53; 2005, p. 335)

Rawls set his principles in an order of priority, meaning that neglecting any of the principles shall never be justified by fulfilling a subsequent principle. Applied to the context of AI, let us formulate the first Rawlsian principle for fair AI with the highest priority as follows:

1. Developers and deployers of an AI system must ensure that the AI system does not threaten the basic liberties of any individual.

Drawing from liberties Rawls himself has listed (Rawls, 1999, p. 53; 2005, p. 335), the guideline is hereby extended with a set of requirements that aim to bring Rawls’s basic liberties into the context of development and application of AI. The first one is:

1.1 AI systems should not endanger but support the freedom of thought and liberty of conscience

Today, AI systems could be used to erode such freedom through surveillance (see Nemitz, 2018; Manheim & Kaplan, 2019; Wirtz et al., 2019; Zuboff, 2019; Véliz, 2021; Coeckelbergh, 2022a; Saura et al., 2022). Recent discussion on technologies that enable mass surveillance^1^ demonstrates the global scope of the threat—even if not necessarily always realised—and how easy it is to mask. The discussion further intensified after the French government passed a regulation allowing AI powered surveillance during Paris 2024 Olympics.^2^ Furthermore, even the mere threat of surveillance or nudging can be seen as a threat to freedom of thought because of how it changes our behaviour to advance someone else’s goals rather than our own (Coeckelbergh, 2022a). Thus, following Rawls’s requirement to prioritise protection of freedom of thought and liberty of conscience, AI ethics guidelines should guide the adoption of AI technologies so that they do not enable mass surveillance, or other types of manipulation or nudging that reduce our autonomy and agency. Considering the priority of the first principle of justice and thus the basic liberties, this should not be done even in the benefit of the least advantaged members of the society.^3^

1.2 AI systems should not compromise but support political liberties and freedom of association, such as the right to vote and to hold public office

This liberty appears problematic since it is tied to democratic processes traditionally defined in national constitutions. AI technologies, on the other hand, are not always limited to national contexts. For example, generative AI systems like chatbots based on Large Language Models (LLMs), as well as AI-enhanced platforms, such as social media, reach a global audience. Ensuring the preservation of political liberties and freedom of association may be easier for national governmental institutions that use AI tools only in national government. For any global organisation, on the other hand, further operationalisation is needed. As such, this is a discussion that would merit its own paper, which is why, in the scope of this paper, I only expand on few key issues of applicability.

As Sen (2010) points out as one of the major difficulties in Rawls’s theory, it is poorly applicable to global contexts and global justice. Although Rawls does introduce a continuation to his theory of justice in *The Law of Peoples* that aims to address the need for international collaboration, it only extends to the context of political liberalism, offering “ideals and principles of the *foreign policy* of a reasonably just *liberal* people” (Rawls, 2001, p. 10) (emphasis by the original author). Its main agents are still peoples, each with their "own internal governments" (Rawls, 2001, p. 3). AI systems are not, however, on the market only for just and liberal people to use. In this situation, one could be drawn towards Rawls’s *historical method* (Rawls, 2005, p. 292–293), to refine the liberties: one could analyse a set of democratic constitutions that exist in the target market of a particular AI technology and seek for best practices that are the most efficient in supporting political liberties and freedom of association. Yet, this would not address the issue that AI systems are not under control by the nation-states—liberal or not—but mostly private companies and/or non-/capped-profits that can operate under several different jurisdictions. Many of these jurisdictions belong to non-democratic—in Rawls’s terms non-decent—governments.

Making sure that AI systems do not compromise but support political liberties and freedom of association is thus challenging and would require extensive international coordination. In Rawls’s view, this would require that the basic institutions of non-liberal societies “meet certain specified conditions of political right and justice and lead its people to honor a reasonable and just law for the Society of Peoples” (Rawls, 2001, p. 60). To do so—assuming optimistically, if not naively, that it would be possible in the first place—would require unforeseen efforts in global politics.

Moreover, freedom of elections is challenged when AI is used to influence potential voters. Candidates can be promoted by interactive social bots that spread messages and communicate with other users in a way that strongly resembles human activity. These bots can be automated either by political campaigners or external sources and can lead to a candidate reaching an unproportionate advantage compared to others that do not have access to these technologies or consider them unacceptable because of their manipulatory nature (see e.g., Brkan, 2019; Coeckelbergh, 2022a, p. 28; see also Kilovaty, 2019; Manheim & Kaplan, 2019). Recent developments seem to have made possible significant increase in efficiency, interactivity, and thus manipulatory potential of such attempts. For instance, popularisation of generative AI technologies has made it considerably easier to produce deepfakes and misleading information, which is profoundly changing digital communication as a basis for political participation (see, e.g., Jones, 2023). This unfortunate potential has been already utilised in parliamentary elections in Europe (e.g., Maker, 2023; Spring, 2024). In Rawlsian terms, the use of AI for such purposes is in principle considered a violation of basic liberties, and hence, unfair. On the other hand, political participation can potentially be enhanced by developing AI tools that facilitate democratic deliberation and encourage political participation (Alnemr, 2020; Bernholz et al., 2021; Helbing, 2021). These elements could thus be highlighted to strengthen this element of basic liberties.

1.3 AI systems should not harm but support the liberty and integrity of the person, including freedom from psychological oppression and physical assault and dismemberment

In the light of recent research (see e.g.,Manheim & Kaplan, 2019; Kilovaty, 2019; König & Wenzelburger, 2020; Laitinen & Sahlgren, 2021; Prunkl, 2022), some of the AI technologies currently in use, such as algorithms for online manipulation and effective deep-fake technologies, are threatening the very autonomy of humans. For example, Kilovaty (2019, p. 469) considers online manipulation to “effectively deprive individuals of their agency by distorting and perverting the way in which individuals typically make decisions.” Similarly, König and Wenzelburger (2020, p. 7) consider this as one of the most serious negative consequences that AI might impose on democracy since freedom and human autonomy are among the core democratic values. Furthermore, Coeckelbergh (2022a, p. 118–122) discusses how AI can be seen through the concept of performance, affecting and conducting our bodies as well as minds. Lastly, AI has already being used in military to automate warfare, which hold several ethical issues with potential to physical oppression and assault (Forrest et al., 2020). For Rawls, such AI systems would be in principle deemed unethical, at least to an extent to which they take us further away from the goal of reaching equal basic liberties for everyone rather than advance it. As shown by, e.g., Johansson (2018), use of autonomous weapons could reduce casualties and thus work in favour of 1.3. in some cases. We have thus once again arrived in a situation where further consideration is needed to contextualise the principle in individual use cases.

1.4 All AI systems should be aligned with the principle of rule of law

Accordingly, AI systems should be used to support, for example, due process and fundamental rights. Current tools adopted by certain judiciary institutions, such as COMPAS used in the US, seem to violate this principle by treating people differently based on, e.g., their ethnicity (on ethical issues concerning AI in jurisprudence, see e.g., Larson et al., 2016). Similarly, the use of AI systems has already led to unlawful arrests of people (see, e.g., Coeckelbergh, 2024, p. 42). These are examples of AI tools that violate the basic liberty of rule of law instead of supporting it, which demonstrates that more consideration is needed in institutions with a key role in ensuring the rule of law when applying AI systems in order for them to align their actions with Rawlsian principles of justice.

One quickly observes that Rawls’s liberties are not free of conflicts. Let us consider, for instance, the persisting conflict between principles 1.1 and 1.3: exercising the freedom from physical assault would benefit from continuous AI-powered mass surveillance to target, e.g., potential terrorist attacks or rapes. This would, however, violate the freedom of thought and liberty of consciousness. The same applies for AI used in military, where the potential harm to the rule of law (1.4) could be estimated minimal compared to the potential positive impact on the liberty and integrity of the person (1.3). Rawls takes note of this issue by stating that basic liberties can be limited “when social circumstances do not allow the effective establishment of these basic liberties”, but only with the one condition: “these restrictions can be granted only to the extent that they are necessary to prepare the way for the time when they are no longer justified” (Rawls, 1999, p. 132). He continues by suggesting that even if such compromises are necessary, the circumstances should still be “sufficiently favorable so that the priority of the first principle points out the most urgent changes and identifies the preferred path to the social state in which all the basic liberties can be fully instituted” (Rawls, 1999, p. 132).

In other words, Rawls’s list of liberties is not absolute – they can and maybe even need to be modified to fit the “social, economic and technological” context of the society under scrutiny (Rawls, 1999, p. 54). He offers, as a response to critique, two methods for defining an appropriate set of basic liberties: 1) a *historical* survey of democratic constitutions to see which ones have traditionally worked well, and 2) an *analytical* consideration based on exhaustion on the set of liberties that are essential for principles agreed upon in the original position (Rawls, 2005, p. 292–293). The second method means developing repeatedly iterations of the list of basic liberties, eventually ending up with one that is fair according to the criteria of the original position. These methods and possible others have been discussed by several academics over the years (see e.g., McLeod & Tanyi, 2021), and could be used to set more refined and contextualised guidelines on the basis of the principles discussed in this paper. Here, the guidelines are drafted with the freedoms listed by Rawls, leaving their further development to future research.

### The Second Principle: Equality of Opportunity and Difference Principle

The second principle is divided into two sub-principles that apply to the “distribution of income and wealth and to the design of organizations that make use of differences in authority and responsibility” (Rawls, 1999, p. 53). First, all social and economic inequalities “must be attached to offices and positions open to all under conditions of fair equality of opportunity” (*equal opportunity principle*) (Rawls, 2005, p. 291). Hence, the basic structure of society must guarantee that each individual has equal opportunities to access the advantageous positions. This is particularly relevant in the context of choices regarding recruitment and performance evaluation, as well as access to education. However, events that influence people’s paths to become eligible for a certain position in the first place also needs to be given attention. For example, a simple recommendation algorithm could influence the choices of people when it comes to skill development or decisions to seek for positions in certain fields (e.g., Kokkodis & Ipeirotis, 2021). When biased, such algorithms could direct some people towards less advantageous positions than others on an unfair basis. This could also lead discrimination of people working certain professions over others – the future of jobs such as freelancer designers and writers has already raised questions of impacts on individuals’ livelihoods and opportunities in life (e.g., Mims, 2024).

Rawlsian guidelines could thus recommend the following:

2. The use and development of AI systems should not negatively impact people’s opportunities to seek income and wealth. If an AI system is used in distribution of advantageous positions, such as recruitment, performance evaluation, or access to education, it needs to be ensured that.
  2.1 the tool is trained with non-biased training data, or appropriate tools are used to mitigate the biases in the final product if no non-biased training data is available (data bias mitigation),
  2.2 the outcome of the use of the tool includes an explanation of the grounds for the outcome it produces (explainability), and.
  2.3 the algorithms used shall encourage neither biased results nor the systematic repetition and amplification thereof in, e.g., the feedback loops of a machine learning system (algorithmic bias mitigation).

If these conditions cannot be met, AI should not be used in the process.

In the light of datasets currently used in such processes, these requirements are likely to be close to impossible to meet. As Russell (2019, p. 129) explains, the bias is often rooted in the culture where the data has been created and extracted. Simply removing the factors that are not supposed to affect the decision—such as race, age, or gender—is not sufficient because of redundant encodings that enable biased prediction from other aspects (Hardt et al., 2016). Although several suggestions have been made about fairness evaluation and correction models for AI datasets (see e.g., Hardt et al., 2016; Mehrabi et al., 2022), there is no evidence implying they were widely adopted. Even if the lack of transparency is not an issue per se and can be challenged as an indispensable value (see, e.g., Holm, 2019), in the context of Rawlsian equality of opportunity, a meaningful level of explainability would be required to avoid systematic biases that constitute a persisting problem in AI-assisted recruitment (e.g., Kazim et al., 2021; Tilmes, 2022).

Yet, one could argue that human decisions are not free of bias, either, and thus using an AI system could lead to decisions that support the equality of opportunity compared to human decision-making (see, e.g., Zerilli et al., 2019). However, from a Rawlsian perspective, the human decision-making should also aim at equality of opportunity, and thus the failure of, e.g., a recruiter to follow the principle should not justify any lesser principle for the use of AI systems. Moreover, AI systems tend to systematise biases towards certain groups of people, which means that the potential to improve the equality of opportunity of some individuals might happen to the detriment of those in particularly vulnerable positions. As we will see below, this can pose a problem also in the light of Rawls’s difference principle. To add to the complexity, for the basic structure of society to ensure people do not lose their livelihoods due to AI would also require extensive measures in, e.g., policy, to ensure changes in labor markets would be appropriately managed and meaningful opportunities found to replace the lost jobs. Therefore, defining the responsibilities between institutions belonging to the basic structure would require more attention, which will be further discussed below.

Characteristic to Rawls’s theory, this principle aims at an ideal situation rather than incremental change, which has deservedly received critique (e.g., Sen, 2010). It also neglects all interpretations of fairness other than Rawls’s, which are frequently discussed amongst researchers of algorithmic fairness (see, e.g., Chouldechova, 2017; Baumann & Loi, 2023; Green, 2022). Consequently, considering the above-discussed need to adjust the list of basic liberties according to their context, deciding upon the biases that should in this ideal situation be mitigated is most likely not universal. Still, as for our experiment following Rawls, we rely on his conception of fairness and maintain that if the use of AI indicates a risk of systematic erosion of equality of opportunity for some individuals or groups thereof, organisations should not use such AI systems in recruitment or other distribution of advantageous positions, no matter the potential increase in efficiency that the tool could bring.

As for the latter part of the second principle, *difference principle*, inequalities “must be to the greatest benefit of the least advantaged members of society” (Rawls, 2005, p. 291). This principle applies to the “distribution of income and wealth and to the design of organizations that make use of differences in authority and responsibility” (Rawls, 1999, p. 53). To apply this principle to the context of AI, the following recommendation is given:

3. All inequalities affected by AI systems, such as acquiring a position of power or accumulation of wealth, must be to the greatest benefit of the least advantaged members of society.

The development of AI technologies has led to concentration of power over many aspects of our lives to tech giants (Coeckelbergh, 2022a; Nemitz, 2018, p. 2–3), which means that the interests of the shareholders of these companies have an increasing influence on the distribution of inequalities, as well as on algorithms that are used to determine the value basis for doing so. Rawls’s difference principle does not merely prohibit advantages detrimental to the least advantaged but requires an *increase* in benefit for the least advantaged as a result of any such inequality, which reflects the maximin principle Rawls uses as the decision theory on choosing one’s action in an event of uncertainty. Accordingly, we should make decisions about the direction of AI development based on an evaluation of the worst possible scenario of each option, and choose an action the worst scenario of which is the least bad. Although Rawls’s use of the maximin principle has been heavily and perhaps deservedly criticised by, e.g., Harsanyi (1975) and further developed by Sen (2010), Rawls persistently defended his version, leading to a continuous discussion notably in the field of economics about its potential to form a basis for fair distribution of resources (e.g., Mongin & Pivato, 2021). Therefore, without going into details of these arguments, Rawls’s perspective is here applied as is.

The current accumulation of wealth from AI that has shown often to harm rather than enhance the lives of the least advantaged (e.g., discriminatory systems used to make life-changing decisions in Tilmes, 2022; AI systems that undermine minorities in, e.g., Bender et al., 2021; Janssen et al., 2022; or AI systems eroding societal structures that protect fundamental rights in Coeckelbergh, 2024), which seems contradictory to the difference principle. However, many of these systems would already be deemed unfair on the basis of principles 1 or 2. Having in mind that Rawls’s principles come in an order of priority, for a system to be considered against the difference principle, it should first be aligned with the preceding principles. For example, an AI system deployed by a financial institution could be used to determine the probability of a prospective lender to fail the contract terms. Even if the system was built in full alignment with the basic liberties and the biases were mitigated appropriately, if the system benefits most the shareholders of the banking institution (and does not, for example, improve the position of the least advantaged in mitigating human biases in loan decisions), it fails in regard to the difference principle.

It is obvious that showing or estimating the implications for the least advantaged in the society in a precise manner is complicated, if not impossible in modern societies. It would first require defining who are the least advantaged, which already poses a great difficulty. To align AI with this principle would thus require active reflection on the impacts of AI systems on different groups of people in both development and deployment. For instance, a company developing a generative, general purpose AI system would need to conduct broad impact analyses and reflect the consequences of their actions as well as their moral duties in a broader scope, which brings them closer to a situation similar to deliberation in an original position.

Therefore, following Rawls’s theory, AI systems should always thus encourage societal improvement when used in processes that lead to inequalities (as opposed to “levelling down” as a result of fairness algorithms, see Mittelstadt et al., 2023). These guidelines would thus require institutions to review their existing approaches, such as data-capitalist business logic for wealth maximization, to align their actions with the Rawlsian principles. Regulating institutions and policy-makers could seek for solutions from regulation that set corresponding minimum requirements for AI systems, as well as standards for enforcement—the soon-to-be-adopted EU AI Act being one (although non-Rawlsian) example of a supranational approach. Private corporations could include the difference principle into their frameworks for innovation so that no system that fails to comply gets to production.

Even then, we would be left with fundamental questions: What kind of balance between state regulation and private power leads to the fairest AI systems overall? How do the measures by private and public institutions tie together into a functional societal order? Will that system still resemble a democracy, which in Rawls’s perspective is the only legitimate form of fair societies? As Coeckelbergh (2024, p. 76) notes, it is not only a question of how much regulation we need but what kind of institutional structure we have in place to implement the policies and principles, and how we change our technologies themselves. Could Coeckelbergh’s technodemocracy (2024, p. 83–83) offer us the answers we are looking for? How about Susskind’s (2022) digital republic or Muldoon’s (2022) platform socialism? Answering these questions would require broader interdisciplinary discussion and policy research that exceed the scope of this paper. As for now, these guidelines offer a reference point for the institutions in the basic structure of society to evaluate the fairness of their development and deployment of AI systems.

Lastly, it is worth noting that due to the context where Rawls created his principles, these guidelines are drafted with liberal democracies in mind, which means that there’s a strong caveat in their universal applicability. This is characteristic to Rawls’s theory of justice and becomes especially apparent in his later developments in *The Law of Peoples* (Rawls, 2001), which has received critique by, e.g., Sen (2010). Now, it has been further illustrated in the application of Rawls’s principles to the context of AI, which indicates a need for further research in ethics perspectives that would better grasp the global nature of AI and thus the need for ethical principles that consider the societal structures in regimes other than democracies, as well.

## Conclusions and Discussion

The goal of this paper has been to experiment with a set of ethics guidelines for AI following John Rawls’s theory of justice as fairness. Today, major ethical concerns of AI technologies are related to inequalities and other issues that threaten the future of democratic governance (see e.g., Brkan, 2019; Coeckelbergh, 2022a, 2022b, 2024; König & Wenzelburger, 2020). Meanwhile, the major AI ethics guidelines introduced so far have shown to neglect the relationship between AI and democracy (see e.g., Hagendorff, 2020) and to lack in disclosing the justifications – the very ethics – behind the principles (Franzke, 2022; Hagendorff, 2020; Jobin et al., 2019). The current paper is an attempt towards strengthening the moral-philosophical basis of the principles that serve as the foundation for applications and operationalisation of ethics amongst both the providers and users of AI systems. This is an exploration of the possibility of creating an ethics guideline that aims at ensuring societal stability and societal structures that support the ethicality of AI. Rawls being one of the most important modern philosophers with the goal of establishing principles of justice for modern democracies, his theory provides a fruitful basis for this exploration. The conclusive set of Rawlsian ethics guidelines for fair AI are:

1. Developers and deployers of an AI system must ensure that the AI system does not threaten the basic liberties of any individual.
  1.1 AI systems should not endanger but support the freedom of thought and liberty of conscience.
  1.2 AI systems should not compromise but support political liberties and freedom of association, such as the right to vote and to hold public office.
  1.3 AI systems should not harm but support the liberty and integrity of the person, including freedom from psychological oppression and physical assault and dismemberment.
  1.4 All AI systems should be aligned with the principle of rule of law.
2. The use and development of AI systems should not negatively impact people’s opportunities to seek income and wealth. If an AI system is used in distribution of advantageous positions, such as recruitment, performance evaluation, or access to education, it needs to be ensured that.
  2.1 The tool is trained with non-biased training data, or appropriate tools are used to mitigate the biases in the final product if no non-biased training data is available (data bias mitigation),
  2.2 The outcome of the use of the tool includes an explanation of the grounds for the outcome it produces (explainability), and.
  2.3 the algorithms used shall encourage neither biased results nor the systematic repetition and amplification thereof in, e.g., the feedback loops of a machine learning system (algorithmic bias mitigation).

If these conditions cannot be met, AI should not be used in the process.

3. All inequalities affected by AI systems, such as acquiring a position of power or accumulation of wealth, must be to the greatest benefit of the least advantaged members of society.

Are these guidelines any better than the ones already drafted? In choosing Rawls’s theory as a basis for the guidelines, I have addressed several issues found in the existing guidelines, the first being previously mentioned as the lack of discussing democracy. These guidelines are also improved in their ability to prioritise: whereas existing guidelines do not offer solutions in cases of conflicting principles (Jobin et al., 2019), Rawlsian guidelines are hierarchical, which helps with resolving priority conflicts. Yet, the analysis shows that a number of conflicts still persist—the Rawlsian approach is not an off-the-shelf solution, or a silver bullet that would eradicate the need for contextualising the principles and exercising active ethical reflection while developing and deploying AI systems, which is, as e.g. Rességuier and Rodrigues (2020) and Heilinger (2022) note, is an essential part of effective application of ethics in the context of AI. To do so, approaches such as those of Rahwan (2018) that highlight the need for public engagement and deliberation could ensure that the principles rooted in rigorous ethics get operationalised in an actionable way, which is a topic for future research.

Furthermore, Jobin et al. (2019) point out that the existing guidelines focused on negative characterisation of ethical values, and thus tended to neglect the possibility of promoting favorable values with AI, not just ensuring the principles are met *despite* of the use of AI in the context in question. In these guidelines, this is considered in guidelines 1.1, 1.2 and 1.3 as well as in the guideline 3, embedded in the difference principle. Taking Rawls’s theory, which is rooted in freedom and equality, as a starting point also addresses what Jobin et al. (2019) identified as another flaw in existing guidelines: they lack in discussing human dignity and solidarity. These two topics are embedded in Rawlsian principles—freedom and equality are fundamental aspects that aim at guaranteeing equal basic liberties for all, and the difference principle guides towards consideration of the least advantaged members of society besides one’s own individual interests. The difference principle also addresses the frequent issue with algorithmic fairness pointed out by Mittelstadt et al. (2023) in their pre-print, according to which strictly egalitarian views of fairness easily lead to “levelling down”, i.e., making everyone worse off. Rawlsian guidelines also offer a philosophically justified ground called for by Franzke (2022), and keep in mind the societal context created by democratic institutions—the lack of which was previously noted by Hagendorff (2020).

What these Rawlsian guidelines are arguably lacking are practical examples and applications in technology, as well as metrics to follow how well the principles are being fulfilled. They are in a high level of abstraction, meaning institutions need to reflect on what they mean in practice in their sectorial context. It must also be noted that these guidelines aim at applying and adapting Rawls’s principles to today’s society that differs in certain essential points from that of the time Rawls initially started working on his theory of justice, which stresses the importance of subjecting the principles to broader public discussion. That is, however, not an issue per se, as drafting principles is only the first part of the process (see e.g., Mittelstadt, 2019; Rességuier & Rodrigues, 2020), and there is currently an active academic discussion around operationalization of AI ethics principles and recommendations (see, e.g., Ayling & Chapman, 2022; Ibáñez & Olmeda, 2022; Bleher & Braun, 2023). The next step would be to subject the guidelines to public deliberation, bring them into practice and test them in real-life conditions, such as those described by Leben (e.g., 2017, 2018) in his work on application of Rawlsian ethics to autonomous vehicles. The principles could be applied by design scientists to find ethically and societally sustainable practices for future AI development, and by practitioners to innovate use cases that are ethically sustainable. The principles could also be tested in efforts to create auditing frameworks for technologies and processes utilising AI technologies, as well as opening a broader dialogue on societal structures that would allow AI development to benefit most the least advantaged. Attaching a list of best practices and further developments would add to the applicability of these guidelines in practice.

One could also question whether subjecting all nations to a Western liberalist normative framework would be desirable or ethical in the first place, as digitalisation has already raised concerns about new forms of colonialist practices (e.g., Adams, 2021; Arora et al., 2023; Couldry & Meijas, 2019; Westerstrand et al., 2024). Rawls’s premise that justice can only be achieved by democratic governments is reflected in the principles (e.g., 1.2.), which would require non-democratic governments to first adopt a new political system in order for them (or the companies operating under their legislature) to produce fair AI systems. This would be unreasonable. It is thus important to see the principles in their normative context and critically discuss their applicability in a global level. Consequently, these guidelines still remain—as is to be expected—at the principle-formation-level, and thus are not alone a solution for ethical AI.

Yet, I hope that these guidelines inspire both academics and practitioners to revisit their own existing guidelines and to evaluate how they relate to the Rawlsian principles. Did they differ in emphases, did they add or omit something? Is there a justification for inclusion or omission of some principles, or values? How about the larger societal context, would your organisation’s guidelines support preservation of societal structures that promote fairness? Even though we need to keep bringing principles into practice, I argue that re-evaluating the moral-philosophical grounds of the existing principles and guidelines is still a vital part of practicing ethics in the dynamic, ever-changing context of AI development.

## Footnotes

1 For example, a recent European Digital Rights (EDRi) report revealed ongoing biometric mass surveillance in several European countries: https://edri.org/our-work/new-edri-report-reveals-depths-of-biometric-mass-surveillance-in-germany-the-netherlands-and-poland/. These technologies are increasingly using AI technologies to collect, process and redistribute data.

2 A piece of news by Reuters (23 March 2023): https://www.reuters.com/technology/france-looks-ai-powered-surveillance-secure-olympics-2023-03-23/.

3 For example, a recent discussion on Apple’s intention to install anti-child porn technology in iPhones has been widely considered a violation of fundamental rights. See, e.g., https://www.pcmag.com/news/report-apple-to-scan-iphones-for-child-sexual-abuse-imagery. It should, therefore, be primarily explored whether there are other measures to protect the integrity of the child (see liberty 1.3) other than digital mass surveillance, so that we would be most likely to follow the “preferred path to the social state in which all the basic liberties can be fully instituted” (Rawls, 1999, p. 132).
